# Physiological and Proteomic Responses of Dairy Buffalo to Heat Stress Induced by Different Altitudes

**DOI:** 10.3390/metabo12100909

**Published:** 2022-09-27

**Authors:** Qin Lan, Zhiyong Cao, Xiujuan Yang, Zhaobing Gu

**Affiliations:** 1 Faculty of Animal Science and Technology, Yunnan Agricultural University, Kunming 650201, China; 2 Faculty of Big Data, Yunnan Agricultural University, Kunming 650201, China; 3 Yunnan Provincial Key Laboratory of Animal Nutrition and Feed Science, Kunming 650201, China

**Keywords:** DIA proteomics, buffalo, altitude, environment stress, adaptation strategies

## Abstract

Buffalo are mainly distributed in low-altitude (LA), medium-altitude (MA), and high-altitude (HA) regions characterised by different thermal and oxygen environments in Yunnan province, China. Due to black skin, sparse hair, and the low density of skin sweat glands, buffalo are more sensitive to heat stress. Here, we used data-independent acquisition (DIA) proteomics to reveal a broad spectrum of proteins that play roles in adaptation to the heat stress of buffalo raised at low altitude or hypoxia at high altitude. LA buffalo showed higher body temperatures than MA- and HA buffalo, and HA buffalo had higher levels of GSH and SOD and lower levels of ROS compared to LA and MA buffalo. In 33 samples, 8476 peptides corresponding to 666 high-confidence proteins were detected. The levels of circulating complement proteins in the immune pathways were lower in LA and MA buffalo than in HA buffalo. There were higher levels of alpha-1 acid glycoprotein in LA buffalo than in MA and HA buffalo. Relative to MA buffalo, levels of blood oxygen delivery proteins were higher in LA and HA buffalo. A higher abundance of apolipoproteins was detected in LA and MA buffalo than in HA buffalo. In summary, buffalo adopted similar adaptation strategies to oxidative stress induced by heat stress or hypoxia, including immunological enhancement, high efficiency of blood oxygen delivery, and the inhibition of lipid oxidation.

## 1. Introduction

Buffalo are the second-largest source of milk globally. Buffalo milk is characterised by high concentrations of proteins, fat, and total solids compared to cow milk [1]. China has rich buffalo resources and produces 5% of the world’s buffalo milk [2]. Yunnan Province is located at altitudes ranging from 76 to 6740 m in Southwestern China and has complex topography. It neighbours Myanmar, Lao, and Vietnam. The geographic distribution of dairy buffalo in Yunnan Province are the states of Dehong, Baoshan, and Dali, which are characterised as low-, medium-, and high-altitude, respectively. There are about 56,000, 8000, and 15,000 buffaloes raised in the three states, respectively. Dehong state, as a typical lowland subtropical area, is characterised by high ambient temperature and humidity in warm seasons, causing chronic heat stress for buffalo in summer [3].

Higher temperatures resulting from global climate change cause low levels of animal production performance, health, and welfare [4,5]. Buffalo are warm-blooded animals, maintaining a constant body temperature of about 38.0 +/−0.5 °C. Due to their black skin, sparse hair, and the low density of skin sweat glands—one-sixth the density of cattle [6]. The optimum temperatures and humidity of buffalo are 13–18 °C and 55–65%, respectively [7], so they are more sensitive to heat stress-induced oxidative stress when the body heat load is greater than heat dissipation. In low-altitude raising regions, high humidity may restrain the efficiency of evaporative heat dissipation in buffalo [8]. Heat stress at low altitudes may adversely affect animal production performance, health, and welfare [9]. In addition, high-altitude raising regions of dairy buffaloes are characterised by a shortage of atmospheric oxygen. High-altitude hypoxia leads to the increased accumulation of reactive oxygen species (ROS) from up-regulation of the mitochondrial electron transport chain and nitric oxide synthase [10,11]. For Chinese Holstein cows, high altitude impairs rumen fermentation and increases the basal metabolism rate [12]. The oxidative stress induced by heat stress and hypoxia isregulated by ROS-mediated activation of hypoxia-inducible factor 1 and heat shock factor 1 [13].

Blood biomarkers are used as indicators of the physiological state of an animal and its responses to changing environmental conditions [14]. We hypothesised that serum proteins related to heat stress-induced oxidative stress at low altitudes or hypoxia-induced oxidative stress at high altitudes would differ in dairy buffalo raised at different altitudes. Proteomics has been widely used to assess the ability of livestock to adapt to heat stress and hypoxia [15]. Specifically, data-independent acquisition (DIA) proteomics, with high reproducibility and high throughput, is a powerful technique in proteomics studies [16]. Here, we used DIA proteomics to reveal differential proteins that play roles in adaptation to heat stress of buffalo raised at low altitude or hypoxia at high altitude. The research findings may be conducive to improving the health and welfare of buffaloes with special feed additives.

## 2. Materials and Methods

### 2.1. Experimental Animals and Management

Dairy buffalo farms used in the experiment were situated at a low altitude of 912.0 m (LA), a medium altitude of 1536.0 m (MA), or a high altitude of 1865.0 m (HA). Thirty-three healthy multiparous (parity = 3.2 ± 1.1) Nili-Ravi × Murrah × Local crossbred buffalo (9, 12, and 12 LA, MA, and HA buffalo, respectively) at mid-lactation (115 ± 10 days) were group-housed in an open-sided barn and fed two times a day using the same total mixed rations (80% whole-plant corn silage ad libitum, 12.5% concentrate feeding, 7.5% corn protein powder) in June. The nutritional values of the three feed ingredients are shown in Supplemental Table S1.

Temperature and humidity loggers (±0.2 °C; Testo 175H1), placed 2.0 m above the floor of the bedding area, recorded the ambient temperature and relative humidity at 30 min intervals to calculate the temperature-humidity index (THI) according to the following equation [17] in the field trails:

THI = (1.8 × T + 32) − (0.55 − 0.0055 × RH) × (1.8 × T − 26)

Here, T is the ambient temperature (°C), and RH is the relative humidity (%).

A waterproof micro-temperature sensor (± 0.5 °C; DS1922L, Wdsen Electronic Technology Co., Ltd. Shanghai, China), fixed in the slot of a T-shaped internal controlled drug release device (without progesterone; DEC International, NZ, Ltd. Hamilton, New Zealand), was placed in the vagina of buffalo to record body temperature at 30 min intervals. At the end of the field trial (15 d), the blood samples were collected with vacutainer tubes via jugular venipuncture. Each sample was centrifuged at 1400× *g* for 10 min to separate the serum. The serum samples were quickly frozen in liquid nitrogen and then stored at −80 °C for later assay.

### 2.2. ELISA Analysis of Serum Parameters

The levels of aldosterone (ALD), glucocorticoid (GC), corticotropin-releasing factor (CRF), prolactin (PRL), glutathione (GSH), insulin-like growth factor 1 (IGF-1), thyroxine 3 (T3), adrenocorticotropic hormone (ACTH), cortisol (COR), superoxide dismutase (SOD), ceruloplasmin (CP), heat shock factor 1 (HSF1), ROS, and hypoxia-inducible factor 1α (HIF-1α) in the serum were measured using ELISA kits according to the manufacturer’s instructions.

### 2.3. Sample Preparation and Fractionation for DDA Library Generation

The serum protein concentrations were determined using a BCA protein assay kit. The inter-rater agreement of all the protein samples was evaluated via an SDS-PAGE gel with Coomassie blue staining. About 20–40 μg of protein from each sample was used for data-dependent acquisition (DDA) library construction. The LA, MA, and HA samples were analysed individually.

The FASP procedure was applied for protein digestion. The protein sample (200 μg) was added to urea buffer (8 M) to a final volume of 60 μL. To this was added dithiothreitol (DTT; 1 M, 0.5 μL), and the mixture was incubated at 56 °C for 30 min. Iodacetamide (25 μL) was then added with stirring, and the resulting mixture was incubated at room temperature for 30 min. NH4HCO3 (100 mM, 240 μL) was added to dilute the urea solution to 1.5 M. To each sample tube, trypsin (4 μg) was added at a protein:trypsin ratio of 1:50 and the resulting mixture was incubated in a 37 °C water bath for 20 h. Formic acid (FA; 10%, 20 μL) was added to the protein mixture and then desalted using a Waters SPE column (Oasis MCX SPE 96-well plate, 186,008,304). Finally, the desalted samples were freeze-dried, and the peptide content was estimated via the UV light spectral density at OD280 (Nanodrop 2000C, Thermo Fisher Scientific, Waltham, MA, USA). Pooled peptides (200 μg) were fractionated into 10 fractions using a High-pH Reversed-Phase Peptide Fractionation Kit (Thermo Scientific™ Pierce™, 848,868).

Each fraction was concentrated by vacuum centrifugation and reconstituted in FA (0.1% *v/v*, 10 µL). The collected peptides (2 μg) were desalted on C18 cartridges (Empore™ SPE Cartridges C18 (standard density), bed ID 7 mm, volume 3 mL, Sigma, Saint Louis, MO, USA) and re-dissolved in FA (0.1% *v*/*v*, 40 µL). An iRT-kit (Ki-3002-2, Biognosys AG, Schlieren, Switzerland) was used to calibrate the relative retention time differences between runs with a 1:3 volume ratio of iRT standard peptides versus the sample peptides.

### 2.4. Data-Dependent Acquisition Mass Spectrometry Assay

Data-dependent acquisitions were used to generate a spectral library to form the query database for the DIA mass spectra in later data analysis. Serum protein samples were fractionated using high pH reversed-phase separation to increase proteomic depth. The gradient elution of the peptides was performed on a homemade column (100 μm × 20 mm, 5 μm-C18). The linear gradient in the column was as follows: 10–30% solution B at 0–97 min, 30–100% solution B at 97–110 min, 100% solution B at 110–120 min and 100% solution B. The peptides were separated using a linear gradient of buffer B (84% acetonitrile and 0.1% FA) at a flow rate of 250 nL min^−1^.

The DDA runs were performed on a Q-Exactive HF mass spectrometer (Thermo Scientific) coupled with an Easy nLC-1200 system (Thermo Scientific) for DDA analysis. Q-Exactive HF mass spectrometry was performed in the data-dependent mode to switch automatically between MS and MS/MS acquisition. The full-scan MS survey was in the positive ion mode, and the scan range was 300–1800 m/z. The mass resolution for MS1 was 60,000 at m/z 200 (3e6 AGC, 200 ms maximum injection time). The resolution for twenty sequential MS2 scans was 30,000 at m/z 200 (3e6 AGC, 120 ms maximum injection time). For MS/MS, the normalised collision energy was set at 27 eV. The DDA spectra were analysed using MaxQuant and were filtered for a false discovery rate (FDR) of 1% at the peptide and protein levels.

### 2.5. Mass Spectrometry Assay for Data-Independent Acquisition

The peptides in each sample were analysed by LC-MS/MS operating in the DIA mode by Shanghai Applied Protein Technology Co., Ltd., Shanghai, China. After the construction of the spectral library for database query, peptide (2 μg) mixed with iRT peptide was injected for DIA mass spectrometry. The Easy nLC-1200 system was used for chromatographic separation using a binary solvent system consisting of solvent A (0.1% FA) and solvent B (84% acetonitrile, 0.1% FA). Solvent A (95%) was used to equilibrate the analytical column (homemade tip column, 75 μm × 250 mm, 1.9 μm-C18). The peptide mixture was separated via gradient elution with a 25 cm tip column at a flow rate of 250 nL.min^−1^ using a linear gradient in the trap column (100 μm × 20 mm, 5 μm-C18; 10–30% solution B at 0–97 min, 30–100% solution B at 97–110 min and 100% solution B at 110–120 min).

HF mass spectrometry (Q-Exactive) was performed in the DIA mode to switch automatically between MS and MS/MS acquisition. The run time was 120 min in the positive ion mode with a linear gradient of buffer B (80% acetonitrile and 0.1% FA) at a flow rate of 250 nL min^−1^. The quality control samples were injected in the DIA mode to monitor the MS performance. DIA scans covered a mass range of 350–1650 m/z with the following settings: SIM full scan resolution 120,000 at 200 m/z, AGC 3e6, 50 ms maximum injection time). Thirty sequential DIA MS2 scans were run at a resolution of 30,000 at 200 m/z, AGC target 3e6, automatic maximum injection time and normalised collision energy of 30 eV.

### 2.6. Mass Spectrometry Data Analysis

MaxQuant software (1.5.3.17) was used to search the FASTA sequence database. The database was downloaded from http://www.uniprot.org, 20 September 2022. The iRT peptide sequence was as follows:

Biognosys|iRT-Kit|Sequence_fusionLGGNEQVTRYILAGVENSKGTFIIDPGGVIRGTFIIDPAAVIRGAGSSEPVTGLDAKTPVISGGPYEYRVEATFGVDESNAKTPVITGAPYEYRDGLDAASYYAPVRADVTPADFSEWSKLFLQFGAQGSPFLK.

The MS search parameters were as follows: cutting enzyme, trypsin; maximum missed cleavages, 2; fixed modification, carbamidomethyl (C); dynamic modifications, oxidation (M) and acetyl (Protein N-term). The reported data were based on 99% confidence for protein identification by false discovery rate (≤1%).

The spectral library was constructed by importing the original raw files, and DDA search results were imported into Spectronaut Pulsar XTM_12.0.20491.4 (Biognosys AG, Schlieren, Switzerland). Spectronaut was used to analyse the DIA data with the above constructed spectral library. The main software parameters were set as follows: retention time prediction type, dynamic iRT; interference on MS2 level correction, enabled; cross run normalisation, enabled. All of the results were filtered based on a Q value cut-off of 0.01 (equivalent to FDR < 1%).

### 2.7. Principal Component Analysis, GO and KEGG Pathway Annotation

The relationship between the serum samples obtained from different altitude buffalo was visualised using a principal component analysis of the quantified proteins using R packages. Significantly differential serum proteins were identified at a fold-change of greater than 1.5 (<0.67) and a *p*-value of less than 0.05. Gene ontology (GO) terms and the Blast2GO software (http://www.blast2go.com/b2ghome, 18 September 2018) were used for the annotation of protein sequences, and the GO annotation results were plotted using R scripts. The differential protein sequences were blasted against the Kyoto Encyclopedia of Genes and Genomes (KEGG) database (http://geneontology.org/, 30 September 2018) to identify KEGG Orthology. Differential proteins were mapped to KEGG pathways.

### 2.8. LC-PRM/MS Quantitative Validation of Targeted Proteins

The levels of serum proteins that are associated with heat stress or hypoxia in buffalo were quantified using parallel reaction monitoring (PRM). Each sample containing peptide (4 μg) was mixed with standard peptide ELGQSGVDTYLQTKSAAGAFGPELSR (200 fmol) after protein digestion. The gradient elution was performed on a C18 HPLC column. Solvent A (95%) was used to equilibrate the analytical column (Thermo Scientific EASY). The peptides were separated using a linear gradient of solvent B (84% acetonitrile and 0.1% FA) at a flow rate of 300 nL min^−1^. The linear gradient in the column was as follows: 5–10% solution B at 0–2 min, 10–30% solution B at 2–45 min, 30–100% solution B at 45–55 min and 100% solution B at 55–60 min.

HF mass spectrometry (Q-Exactive) was performed in the PRM/MS acquisition mode. Full-scan MS surveys (m/z 300–1800) were acquired with a mass resolution (R) of 60,000 at m/z 200 (3e6 AGC 200 ms maximum injection time). This was followed by twenty sequential MS2 scans for higher-energy collisional dissociation (HCD) that conformed to the following parameters: isolation window, 1.6 Th; R, 30,000 at m/z 200; AGC, 3e6; maximum IT, 120 ms; MS2 activation type, HCD. For MS/MS, the normalised collision energy was 27. Skyline v3.5.0 was used to analyse the PRM raw data. The peptide settings used for the Skyline import were consistent with the MaxQuant search parameters (i.e., enzyme set to trypsin, max missed cleavages set to 2). Three consecutive high-intensity peptides were selected to import into Skyline for each set of Skyline analyses. The number of targeted proteins, the sequences of the targeted peptides, the charges of the parent ions, and the peak areas were determined. The total peak areas were calibrated against internal peptide standards using heavy isotopes, and the target peptides were quantified based on the total peak areas.

## 3. Results

### 3.1. Physiological Parameters of Buffalo Raised at Different Altitudes

**Figure 1:**
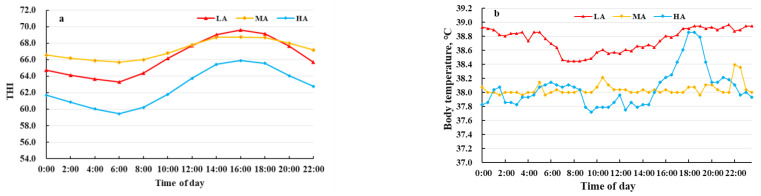
Rhythms of vaginal temperature for dairy buffaloes. (**a**) temperature humidity indexes (THI), (**b**) body temperatures (measured vaginally). LA, MA and HA indicated buffaloes raised at low, medium, and high altitude, respectively.

**Figure 2:**
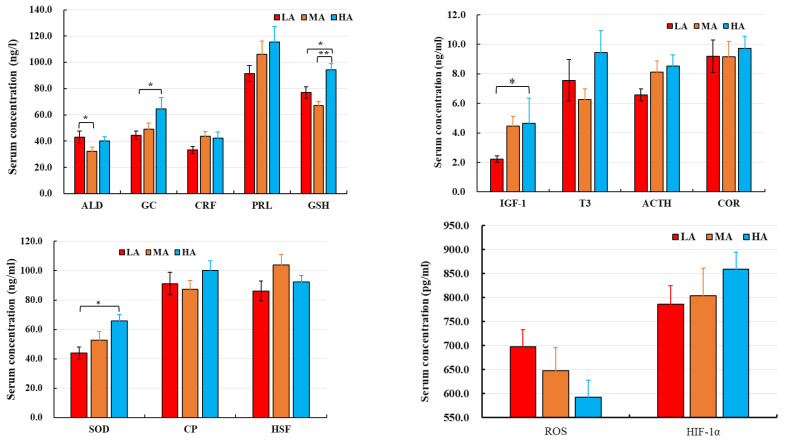
Serum parameters of dairy buffaloes raised at different altitudes. LA, MA, and HA are indicated buffaloes raised at low, medium, and high altitude, respectively. *p < 0.05, and **p < 0.01.

The THI values and buffalo body temperatures are shown in Figure 1. The THI values of LA and MA buffalo (66.3 and 67.2, respectively) were higher than for HA buffalo (62.6). LA buffalo had significantly higher body temperature (38.8 °C) than MA and HA buffalo (38.0 and 38.1 °C, respectively). No significant differences were observed in the serum levels of CRF, PRL, T3, ACTH, COR, CP, HSF, ROS, and HIF-1α in buffalo raised at different altitudes. In contrast, HA buffalo had higher levels of IGF-1, GSH and SOD and lower levels of ROS as compared to LA and MA buffalo (Figure 2).

### 3.2. Spectral Library Construction of DIA Proteomics

**Figure 3:**
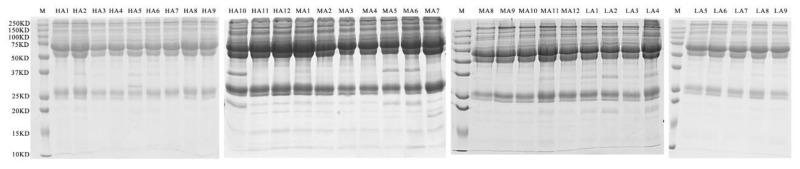
SDS-PAGE of the serum proteins in LA, MA, and HA buffaloes. M, protein marker (10–250 kD); LA, MA, and HA are indicated buffaloes raised at low, medium, and high altitude, respectively.

The SDS-PAGE gel assay with Coomassie blue staining indicated that the proteins had good consistency in the serum samples (Figure 3). The BCA protein assay showed differences in the amount of total protein (10.36, 10.44 and 11.82 μg μL^−1^ for LA, MA, and HA buffalo, respectively), so the same amount of protein was used for further analyses of all the samples (Supplemental Table S2). The samples were digested with the in-solution digestion method and identified using the DIA proteomic technique. The DDA library from Maxquant 1.5.3.17 had 509 protein groups and 3990 peptides, while the DIA library from Spectronaut Pulsar 12.0.20491.4 had 535 protein groups and 3790 peptides. When these libraries were merged, the resulting spectral library had 749 protein groups and 5388 peptides.

### 3.3. Data Reliability and Principal Component Analysis

**Figure 4:**
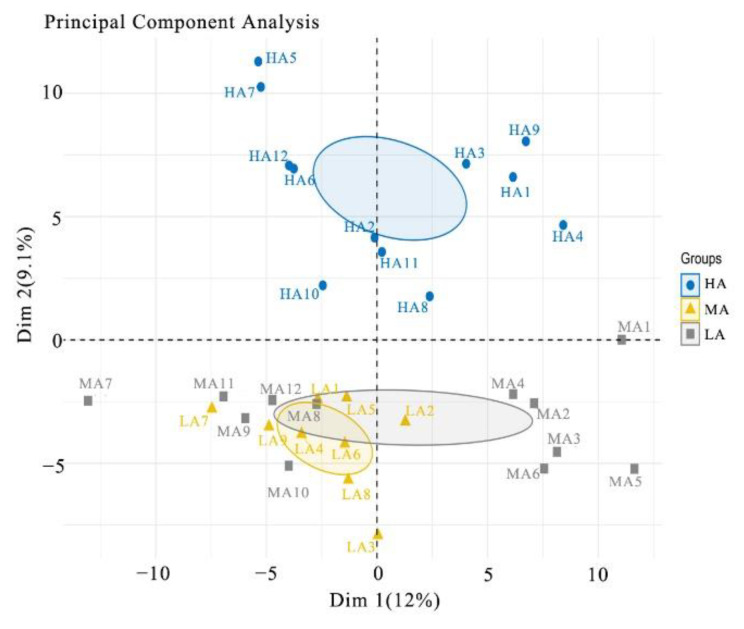
Principal component analysis of proteome profiles. LA, MA, and HA samples are marked with yellow triangle, gray square, and blue circle, respectively. LA, MA, and HA are indicated buffaloes raised at low, medium, and high altitude, respectively.

Spectronaut™ Pulsar X was used to evaluate the correlations in the quantitative data from the protein expression analyses. During data acquisition, there was an average of 7.4 data points per chromatographic peak, which was adequate for accurate quantification. The average peak capacity was 503.2, reflecting the highly efficient chromatographic separation achieved by the DIA analysis. The elution times of the 11 standard peptides indicated the high stability of the chromatographic analysis. False-positive proteins were filtered at a 1% protein FDR threshold so that only proteins with high credibility were kept and all of the average CScore values were greater than 1. The principal component analysis of the quantified proteins provided a visualisation of the relationships between the samples in this study. The biological replicates clustered appropriately and could be distinguished to display the dataset with reasonable reproducibility (Figure 4).

### 3.4. Differential Protein Identification

There were 8476 peptides identified in the serum, which corresponded to 666 high-confidence proteins in the Spectronaut Pulsar X search engine. The results from the protein quantitative analyses and differential expression analyses are shown in Supplemental Table S3. Analysis of variance revealed 60 proteins that were relevant to adaptation to environmental stresses that were significantly differentially abundant in the serum of buffalo raised at different altitudes (Table 1). These differentially expressed proteins in the serum were selected for further analysis based on the fold-change (≥1.50 or <0.67) and *p*-value < 0.05 (Supplemental Table S3). The serum proteins were displayed in a volcano plot (Supplemental Figure S1), and hierarchical clustering showed they could be grouped according to their differential abundance (Supplemental Figure S2). Functional analysis showed that these differentially expressed proteins were involved in immunoregulation, lipoprotein metabolism, the regulation of blood coagulation, oxygen binding and delivery, and antioxidant stresses (Table 2).

### 3.5. Gene Ontology Analysis

The GO terms that were highly enriched in the 60 differentially expressed proteins were identified by DIA proteomics (Table 2). In a pairwise comparison of LA and MA buffalo, 10 GO terms were mainly related to major histocompatibility complex (MHC) protein, oxygen binding and delivery. Eight GO terms were mainly and significantly related to oxygen binding and delivery in a pairwise comparison of MA and HA buffalo. In another pairwise comparison of LA and HA buffalo, 20 GO terms were significantly related to the regulation of immune cells, lipoprotein lipid oxidation, blood coagulation and protein ubiquitination. In the three pairwise comparisons (LA vs. MA, MA vs. HA, and LA vs. HA), buffalo shared two GO terms: GO 0002376-immune system process and GO 0050896-response to environmental stress (Supplemental Figure S3, Supplemental Table S4).

### 3.6. Quantitative Analysis of Targeted Proteins with Parallel Reaction Monitoring

Twenty-five of the 60 differentially expressed proteins from the DIA analyses relating to immunoregulation, lipoprotein metabolism, and the regulation of blood coagulation were selected for PRM analysis. Based on the identification of peptides for which FDR < 30%, 12 target peptides of seven proteins were selected for quantitative PRM analysis. As shown in Table 3, there were 2.06- and 2.48-fold changes in the levels of alpha-1 acid glycoprotein (D2U6V0) in LA buffalo compared to MA and HA buffalo, respectively (*p* < 0.05). No significant differences were detected in the levels of lipoprotein lipase (E1ACW2) and beta-casein (B7VGH4) between LA and HA buffalo, but there were significantly higher levels of both proteins in LA and HA buffalo compared to MA buffalo (*p* < 0.05). The levels of apolipoprotein C-III (Fragment; L8IHQ3) and kappa-casein (A0A1L6BP00) were significantly higher in LA buffalo than in MA buffalo (*p* < 0.01), but there were no differences in the two pairwise comparisons of LA and HA buffalo or MA and HA buffalo.

## 4. Discussion

### 4.1. Blood Physiological Parameters of Buffalo

The temperature–humidity index is widely used to evaluate the level of heat stress [18], with 72 being the threshold for dairy cows [3]. The threshold of heat stress, however, may be lower than 72 for dairy buffalo due to black skin, sparser hair, and fewer sweat glands compared to dairy cows [6,19]. LA buffalo may suffer from chronic heat stress, causing oxidative stress with an imbalance between oxidants and antioxidants. To reduce heat load from the ambient environment with the decrease in temperature gradient between internal organs and external environment [20], LA buffalo had significantly higher body temperature than MA and HA buffalo.

ROS at low levels play important roles in immune function and redox regulation [21], but excessive ROS that are not scavenged under long-term heat or oxidative stress induce lipid peroxidation and oxidative damage to protein [22,23,24]. SOD and GSH are important cellular antioxidants for scavenging ROS. In our study, increased production of ROS was detected, which suppressed the levels of serum antioxidants [25]. Lower levels of GSH and SOD were present in LA buffalo as compared to MA and HA buffalo. HA buffalo live in a comfortable thermal environment (17.5 °C and THI 62.6) and had higher levels of GSH than the LA and MA buffalo. HA buffalo also showed higher serum levels of HIF-1α, but this increase was not significantly different compared to LA and MA buffalo.

### 4.2. Blood Immunoglobulins of Buffalo

Heat stress suppresses the immune function and antioxidant capacity of farm animals [26,27], and increased susceptibility to diseases was observed in heat-stressed animals [28]. Complement pathways and blood coagulation are important natural antibacterial systems [29], and complement proteins play important roles in the elimination of pathogens with complement pathways [30]. We detected that the serum levels of immunoglobulins, including complement factor H, complement component C8 alpha chain, complement factor I, complement factor H-related protein 3 (Fragment), complement component C6, complement factor B, complement C5, and properdin were lower in LA and MA buffalo than in HA buffalo (Table 1). Complement factor H is a member of the complement regulator and plays an important role in complement activation pathways [31].

Most of the serum immunoglobulins were at lower levels in LA buffalo than in MA and HA buffalo, which indicated that buffalo raised at low altitudes suffered from heat stress. Alpha-1 acid glycoprotein, an acute-phase protein produced by the liver and peripheral tissues, which plays a role in immune system regulation [32], was at higher levels in LA buffalo than in MA and HA buffalo. Beta-casein, which regulates the innate and adaptive immune responses in ruminants [33], showed higher levels in LA buffalo than in MA and HA buffalo. Selenoprotein P plays an important role in the transportation of selenium and is one of the main blood antioxidants [34], and glutathione peroxidase is also an important antioxidant [35,36,37]. Both selenoprotein P and glutathione peroxidase were at higher levels in LA and HA buffalo than in MA buffalo. It was inferred that the impact of oxidative stress resulting from heat stress at low altitude or hypoxia at high altitude on the immune responses of buffaloes could be mediated with high levels of serum antioxidants.

### 4.3. Blood Oxygen–Delivery Proteins of Buffalo

As a metalloprotein and the most abundant haem-containing protein in red blood cells, haemoglobin is responsible for blood oxygen delivery. Haemoglobin subunit alpha-1, haemoglobin subunit alpha-2, haemoglobin foetal subunit beta, haemoglobin subunit beta-A, haemoglobin subunit alpha-I/II and haemoglobin beta were present at higher levels in LA and HA buffalo than in MA buffalo. Unexpectedly, there were no differentially abundant proteins or significantly enriched GO terms related to blood oxygen delivery when comparing LA and HA buffalo (Table 2). Buffalo raised at low altitudes with high ambient temperature and relative humidity in summer may suffer from chronic heat stress, and HA buffalo adapt to hypoxia with highly efficient blood oxygen delivery. Heat stress induces local tissue hypoxia due to blood flow redistribution for increased skin heat dissipation [38,39,40], and it was reasonable that no significantly enriched GO terms related to blood oxygen delivery were detected between LA and HA buffalo.

HA buffalo showed higher levels of haemogen, haemopexin, lactoferrin, and serotransferrin, which possibly increased the amount of blood oxygen to meet the physiological demands. HA buffalo also had higher levels of coagulation factors V and IX compared to LA buffalo. Kallikrein and plasminogen were at higher levels in HA buffalo than in LA buffalo, which have roles in the renin-angiotensin system by converting prorenin into renin and in blood coagulation [41].

Vitamin K-dependent protein S, which circulates in the blood, functions as a cofactor and is important for the inactivation of coagulation factors V and VIII on membrane surfaces [42]. Buffalo raised at high altitudes had higher levels of vitamin K-dependent protein S with anticoagulant properties [43,44], and this higher abundance of vitamin K-dependent protein S may play a role in increasing blood flow to meet the blood oxygen supply of dairy buffalo raised at high altitudes. Heat stress induces oxidative stress and hypoxia [39]. To satisfy the requirements of oxygen delivery for internal organs, an increase in blood flow is necessary for dairy buffalo under chronic heat stress. A higher abundance of vitamin K-dependent protein Z was detected in LA and HA buffalo compared to MA buffalo. As a cofactor, vitamin K-dependent protein Z inhibits coagulation factor Xa on phospholipid surfaces to avoid thrombosis and the coagulation response, thereby promoting smooth blood flow [45]. This indicated that buffalo adapt to hypoxia at high altitudes and heat-stress-induced hypoxia at low altitudes through a high blood oxygen delivery capacity.

### 4.4. Serum Apolipoproteins of Buffalo

Apolipoproteins are involved in the combination and transportation of lipids [46]. A lower abundance of apolipoprotein C-IV and apolipoprotein F (Fragment) was detected in LA and HA buffalo than in MA buffalo. The trend for phospholipid transfer protein and proactivator polypeptide, which are involved in lipoprotein metabolism and the regulation of lipid metabolic processes, was similar [47]. However, HA buffalo were higher levels of apolipoprotein D, which regulates the protection from oxidative stress and increases stress resistance [46,48]. The HA buffalo also had higher levels of vitamin D binding protein than LA and MA buffalo. Vitamin D binding proteins are the major carriers of vitamin D [49,50], which is involved in the regulation of fatty acid β-oxidation and energy metabolism [51], inhibiting the activation of stress-activated protein kinases [52].

## 5. Conclusions

Our study indicated that low-altitude buffalo adapted to heat stress-induced oxidative stress with high levels of alpha-1 acid glycoprotein and apolipoproteins, whereas high-altitude buffalo adapted to hypoxia-induced oxidative stress with high complement protein levels for immunological enhancement. In summary, buffalo adopted similar adaptation strategies to oxidative stress induced by heat stress or hypoxia with high levels of serum antioxidants, immunological enhancement, the inhibition of lipid oxidation, and high efficiency of blood oxygen delivery. The research findings may be beneficial to improve the health and welfare of buffaloes with feed additives that enhance immune function and cutaneous circulation.

## Figures and Tables

**Table 1:** Distinct serum proteins of buffaloes raised at different altitudes.

Protein ID	Protein Name	LA	MA	HA
Q28085	Complement factor H	5,911,034.4 ^b^	5,846,954.6 ^b^	8,003,723.2 ^a^
L8I2Z1	Complement component C8 alpha chain	822,025.5 ^b^	916,592.3 ^b^	1,185,262.4 ^a^
L8IHJ7	Complement factor I	5,073,044.5 ^b^	5,423,548.3 ^b^	6,754,342.4 ^a^
L8IE64	Properdin (Fragment)	520,308.3 ^b^	518,567.5 ^b^	669,974.8 ^a^
L8IGE2	Complement factor H-related protein 3 (Fragment)	43,900.4 ^b^	38,432.8 ^b^	67,400.8 ^a^
Q2UVX4	Complement C3	23,107,853.3 ^ab^	21,185,578.8 ^b^	26,465,504.0 ^a^
F1MM86	Complement component C6	704,760.6 ^ab^	642,334.4 ^b^	911,150.4 ^a^
P81187	Complement factor B	9,508,441.3 ^b^	10,024,967.0 ^ab^	11,490,473.8 ^a^
L8J0E1	Complement C5	1,908,360.9 ^b^	2,204,806.2 ^a^	2,240,134.3 ^a^
P17697	Clusterin regulation of complement activation	997,871.7 ^b^	1,381,123.9 ^a^	1,311,446.6 ^a^
L8HKR7	Ig gamma-3 chain C region (Fragment)	122,983,076.8 ^b^	272,235,860.9 ^ab^	287,926,333.6 ^a^
L8HP94	Ig alpha-1 chain C region (Fragment)	63,903.4 ^ab^	78,224.1 ^a^	55,975.5 ^b^
Q7M3F3	Alpha-lactalbumin	10,190.8 ^a^	3491.5 ^b^	9000.8 ^ab^
V5RKP7	Beta lactoglobulin (Fragment)	23,405,971.6 ^ab^	47,755,768.4 ^a^	8,275,413.5 ^b^
C3W955	Beta-lactoglobulin	184,142.0 ^a^	66,021.5 ^b^	118,728.6 ^ab^
F1MHC3	CD44 antigen	70,788.3 ^a^	57,362.6 ^ab^	44,820.5 ^b^
B7VGH4	Beta-casein	310,429.6 ^a^	113,776.7 ^b^	147,863.8 ^ab^
K9ZS80	K-casein (Fragment)	24,306.4 ^a^	11,099.1 ^ab^	7056.9 ^b^
A0A1L6BP00	Kappa-casein	24,079.1 ^ab^	50,215.8 ^a^	19,399.3 ^b^
Q3YJI8	MHC class I antigen (Fragment)	459,090.4 ^b^	1,910,361.4 ^a^	1,304,512.7 ^ab^
D2U6V0	Alpha-1 acid glycoprotein	6,132,936.6 ^a^	4,486,339.8 ^ab^	3,510,769.9 ^b^
Q2KJF1	Alpha-1B-glycoprotein	6073654.1 ^a^	3,382,706.8 ^b^	5,714,935.8 ^ab^
I6SJ59	Selenoprotein P	39,427.8 ^a^	16,819.0 ^b^	25,330.5 ^a^
F8UTU9	Glutathione peroxidase (Fragment)	152,540.5 ^a^	76,484.9 ^b^	117,009.8 ^ab^
A2VE03	ITCH protein	13,480,011.6 ^b^	10,037,581.9 ^b^	70,279,200.0 ^a^
A7MBB0	VCAM1 protein	53,086.1 ^ab^	34,630.1 ^b^	70,988.3 ^a^
L8J552	Microfibril-associated glycoprotein 4 (Fragment)	6731.8 ^b^	19,358.0 ^a^	12,106.7 ^b^
F1MKS5	Histidine-rich glycoprotein	3,266,560.6 ^a^	2,577,096.9 ^b^	2,840,214.9 ^ab^
L8IJH2	Leucine-rich alpha-2-glycoprotein	3,057,520.8 ^a^	2,452,175.9 ^b^	2,764,774.9 ^ab^
G1K1X9	Vitamin K-dependent protein Z	445,107.1 ^a^	421,009.4 ^b^	351,618.1 ^a^
L8IQM7	Vitamin K-dependent protein S	795,398.2 ^b^	792,676.4 ^b^	921,083.0 ^a^
Q3MHN5	Vitamin D-binding protein	7,839,167 ^ab^	7,718,252.2 ^b^	9,062,119.1 ^a^
E1BJN3	Amine oxidase	83,957.7 ^ab^	78,938.2 ^b^	134,389.1 ^a^
L8HRL7	Amine oxidase (Fragment)	179,222.3 ^ab^	150,927.4 ^b^	243,629.0 ^a^
L8HTY4	Amine oxidase (Fragment)	2,291,499.2 ^b^	2,849,165.4 ^a^	2,660,405 ^ab^
Q29437	“Primary amine oxidase, liver isozyme”	757,429.0 ^b^	1,052,047.6 ^a^	973,569.0 ^a^
L8IJ46	Serum amyloid A protein	264,612.5 ^b^	484,433.4 ^a^	411,107.2 ^ab^
L8IP29	Hepatocyte growth factor activator	44,423.2 ^b^	48,712.5 ^b^	59,188.6 ^a^
L8IUV1	Hepatocyte growth factor-like protein	82,650.3 ^b^	73,035.3 ^b^	100,062.1 ^a^
F1MMK9	Protein AMBP	3,690,957.5 ^ab^	3,457,769.0 ^b^	4,323,694.5 ^a^
A8E196	Adult beta-globin	7,859,566.5 ^a^	1,420,398.8 ^b^	5,154,006.6 ^ab^
F1MDA4	Hemogen	153,398.1 ^b^	145,803.7 ^b^	210,144.2 ^a^
D4QBF4	Haemoglobin beta	373,158.7 ^a^	36,534.2 ^b^	132,260.7 ^b^
P04346	Haemoglobin subunit beta-A	638,496.4 ^a^	159,891.8 ^b^	544,179.7 ^a^
P09423	Haemoglobin subunit alpha-I/II	8,726,628.5 ^a^	1,434,904.3 ^b^	4,482,615.4 ^ab^
Q9TSN7	Haemoglobin subunit alpha-1	1,123,791.6 ^a^	193,289.1 ^b^	878,525.5 ^ab^
Q9TSN8	Haemoglobin subunit alpha-2	7,920,238.3 ^a^	1,293,871.4 ^b^	5,010,340.8 ^a^
L8HVY9	Hemopexin	17,375,065.6 ^ab^	17,231,204.0 ^b^	22,307,675.4 ^a^
G9DAR3	Lactoferrin	44,437,656.0 ^b^	51,004,339.7 ^b^	62,055,141.0 ^a^
L8HWR0	Serotransferrin	78,140,829.8 ^b^	90,942,537.7 ^ab^	104,291,760.7 ^a^
L8IA26	Plasminogen (Fragment)	2,852,684.3 ^ab^	2,415,660.0 ^b^	3,391,240.3 ^a^
Q2KJ63	Plasma kallikrein	472,927.9 ^b^	461,005.8 ^b^	609,854.1 ^a^
F1MSZ6	Antithrombin-III	3,855,682.9 ^a^	3,194,585.3 ^ab^	2,450,037.3 ^b^
L8I612	Coagulation factor V	79,479.8 ^b^	97,967.3 ^a^	93,141.4 ^ab^
L8IH10	Coagulation factor IX	402,115.3 ^b^	477,317.8 ^a^	469,924.1 ^a^
L8IA55	Apolipoprotein C-IV	498,024.1 ^ab^	644,042.3 ^a^	361,942.8 ^b^
L8I3W2	Apolipoprotein F (Fragment)	160,853.6 ^ab^	238,038.9 ^a^	105,477.5 ^b^
F1MS32	Apolipoprotein D	845,238.8 ^b^	877,878.2 ^b^	1,551,254.0 ^a^
Q58DL9	Phospholipid transfer protein	60,817.3 ^ab^	71,999.2 ^a^	53,606.6 ^b^
L8IXW5	Proactivator polypeptide	10,898.0 ^b^	36,197.7 ^a^	20,660.7^ab^
F1MNM2	Phosphatidylinositol-glycan-specific phospholipase D	46,791.9 ^ab^	59,825.0 ^a^	44,146.0 ^b^

Data are the MS2 peak area of proteins; ANOVA statistical test was used to acquire distinct proteins; LA, buffalo at low altitude; MA, buffalo at medium altitude; HA, buffalo at high altitude; Row values with different lowercase superscripts ^a^, ^b^ are significantly different at *p* < 0.05.

**Table 2:** GO enrichments of the differentially abundant proteins.

GO_ID	Gene Ontology Terms	Protein ID	*p*-Value
LA vs.MA			
0005344	Oxygen carrier activity	A8E196, D4QBF4, P04346, P09423, Q9TSN7, Q9TSN8	3.25 × 10^−7^
0019825	Oxygen binding		
0140104	Molecular carrier activity		9.39 × 10^−7^
0020037	Heme binding		2.78 × 10^−5^
0046906	Tetrapyrrole binding		6.87 × 10^−5^
0005833	Haemoglobin complex		3.25 × 10^−7^
0005215	Transporter activity		0.003
0005506	Iron ion binding	P09423, Q9TSN7, Q9TSN8	0.038
0042612	MHC class I protein complex	Q3YJI8	0.046
0042611	MHC protein complex		
MA vs. HA			
0019825	Oxygen binding	A8E196, D4QBF4, P04346, P09423, Q9TSN7, Q9TSN8	6.38 × 10^−9^
0005344	Oxygen carrier activity		
0140104	Molecular carrier activity		2.76 × 10^−8^
0020037	Heme binding		2.15 × 10^−6^
0046906	Tetrapyrrole binding		6.62 × 10^−6^
0005215	Transporter activity		0.005
0005833	Haemoglobin complex		6.38 × 10^−9^
0005506	Iron ion binding	P09423, Q9TSN7, Q9TSN8	0.044
**LA vs. HA**			
0032682	Negative regulation of chemokine production	F1MS32	0.038
0032642	Regulation of chemokine production		
0071637	Regulation of monocyte chemotactic protein-1 production		
0032602	Chemokine production		
0071605	Monocyte chemotactic protein-1 production		
0002686	Negative regulation of leukocyte migration		
2000402	Negative regulation of lymphocyte migration		
2000405	Negative regulation of T cell migration		
0034439	Lipoprotein lipid oxidation		
0060588	Negative regulation of lipoprotein lipid oxidation		
0060587	Regulation of lipoprotein lipid oxidation		
0006629	Lipid metabolic process	L8IXW5, A6QLW1, F1MS32	0.036
0033554	Cellular response to stress	A6QLW1, A2VE03	0.042
0003796	Lysozyme activity	A0A0C5ADR0	0.038
2000266	Regulation of blood coagulation	F1MSZ6	
0019787	Ubiquitin-like protein transferase activity	A2VE03	0.038
0061659	Ubiquitin-like protein ligase activity		
0090085	Regulation of protein deubiquitination		

LA, buffalo at low altitude; MA, buffalo at medium altitude; HA, buffalo at high altitude.

**Table 3:** Quantification results of DIA and PRM in buffalo serum proteins.

			DIA Results			PRM Results	
Protein ID	Protein Name	LA	MA	HA	LA	MA	HA
D2U6V0	Alpha-1 acid glycoprotein	6,132,936.6 ^a^	4,486,339.8 ^a^	3,510,769.9 ^b^	0.01275 ^a^	0.00619 ^b^	0.00515 ^b^
F1MSZ6	Antithrombin-III	3,855,682.9 ^a^	3,194,585.3 ^a^	2,450,037.3 ^b^	0.07293	0.11303	0.08698
E1ACW2	Lipoprotein lipase	172,129.6 ^ab^	271,667.7 ^a^	79,035.1 ^b^	0.1019 ^a^	0.03868 ^b^	0.08046 ^a^
B7VGH4	Beta-casein	310,429.6 ^a^	113,776.7 ^b^	147,863.8 ^b^	7.50464 ^a^	4.04234 ^b^	6.80145 ^a^
L8IHQ3	Apolipoprotein C-III (Fragment)	10,570,906.6 ^a^	14,212,911.3 ^a^	8,912,108.0 ^b^	0.07345 ^a^	0.04655 ^b^	0.05991 ^ab^
A0A1L6BP00	Kappa-casein	24,079.1 ^b^	50,215.8 ^a^	19,399.3 ^b^	9.73609 ^a^	5.85045 ^b^	7.12996 ^ab^

DIA results are the MS2 peak area of proteins, and PRM results are the peak area of target peptide segments after internal standard correction; Row values with different lowercase superscripts ^a^, ^b^ are significantly different at *p* < 0.05.

## Data Availability

The data presented in this study are available in article and Supplementary materials.

## Supplementary Materials

The following supporting information can be downloaded at: https://www.mdpi.com/article/10.3390/metabo12100909/s1, Figure S1: Volcano plot of the proteins identified as differentially abundant in the three pairwise comparisons of serum; Figure S2: Hierarchical clustering of serum proteins identified as differentially abundant; Figure S3: Pairwise comparisons of the gene ontologies (GO) of the serum proteins between the three farms; Table S1: Nutrient content of feed samples (%); Table S2: The same amount of proteins (μg) with different volumes (μl) for further analysis; Table S3 Protein identification and Protein quantitative; Table S4 Buffalo share two common GO enrichment.
