# Supplementary material for: Physiological and Proteomic Responses of Dairy Buffalo to Heat Stress Induced by Different Altitudes
Source: Metabolites. 2022 Sep 27;12(10):909. doi: 10.3390/metabo12100909 (PMC9609643; doi:10.3390/metabo12100909)
Supplement: Supplementary file 1 [file metabolites-12-00909-s001.zip › Supple figures.pdf]

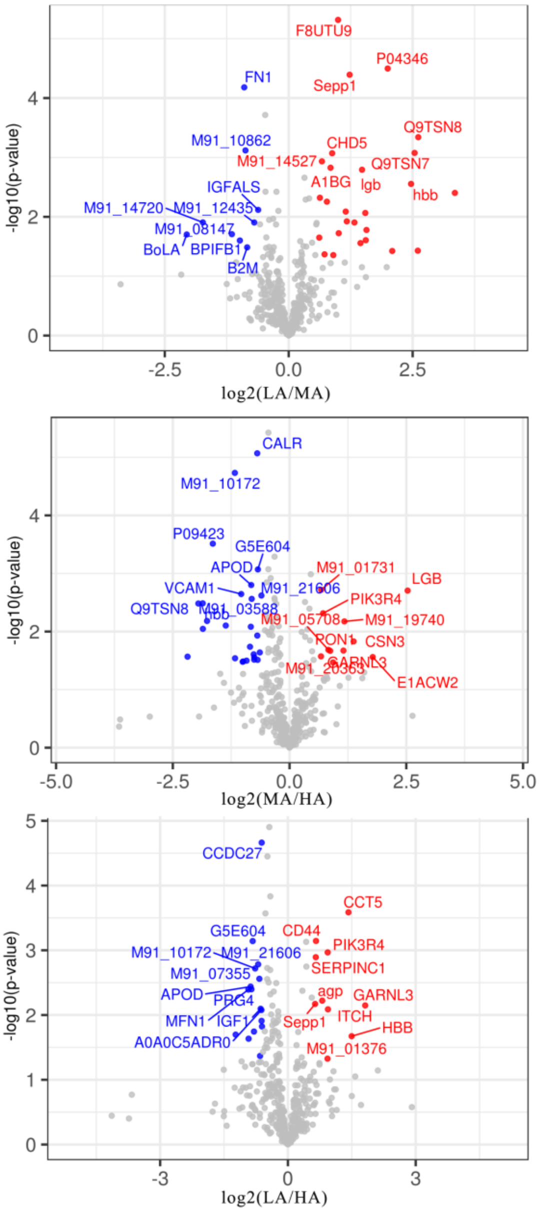

Figure. S1 Volcano plot of the proteins identified as differentially abundant in the three pairwise comparisons of serum. Each point represents one protein. Red and blue dots represent up- and down-changed proteins in serum abundance, respectively. LA, buffalo at low altitude; MA, buffalo at medium altitude; HA, buffalo at high altitude.

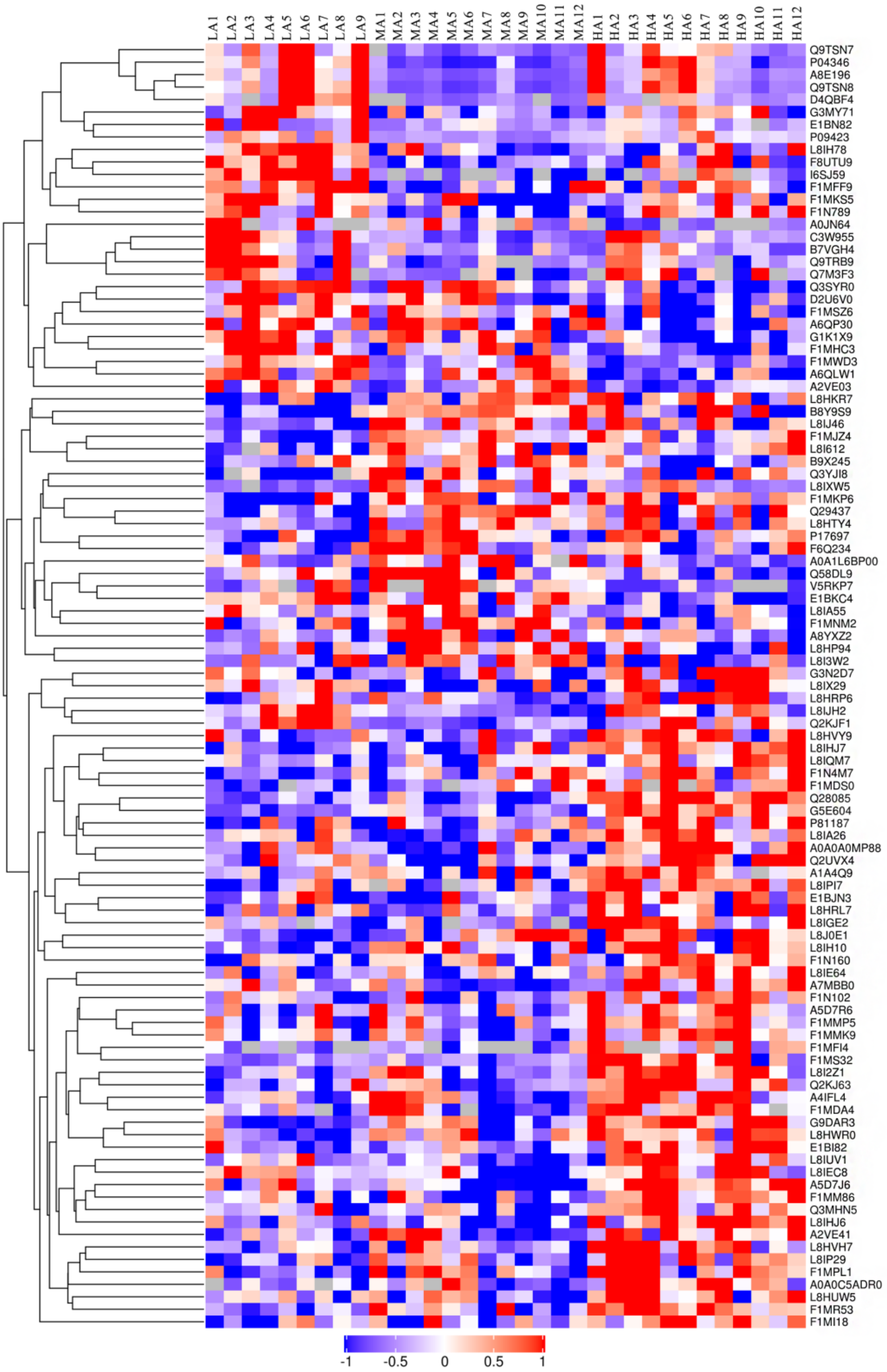

Figure. S2 Hierarchical clustering of serum proteins identified as differentially abundant. LA, buffalo at low altitude; MA, buffalo at medium altitude; HA, buffalo at high altitude.

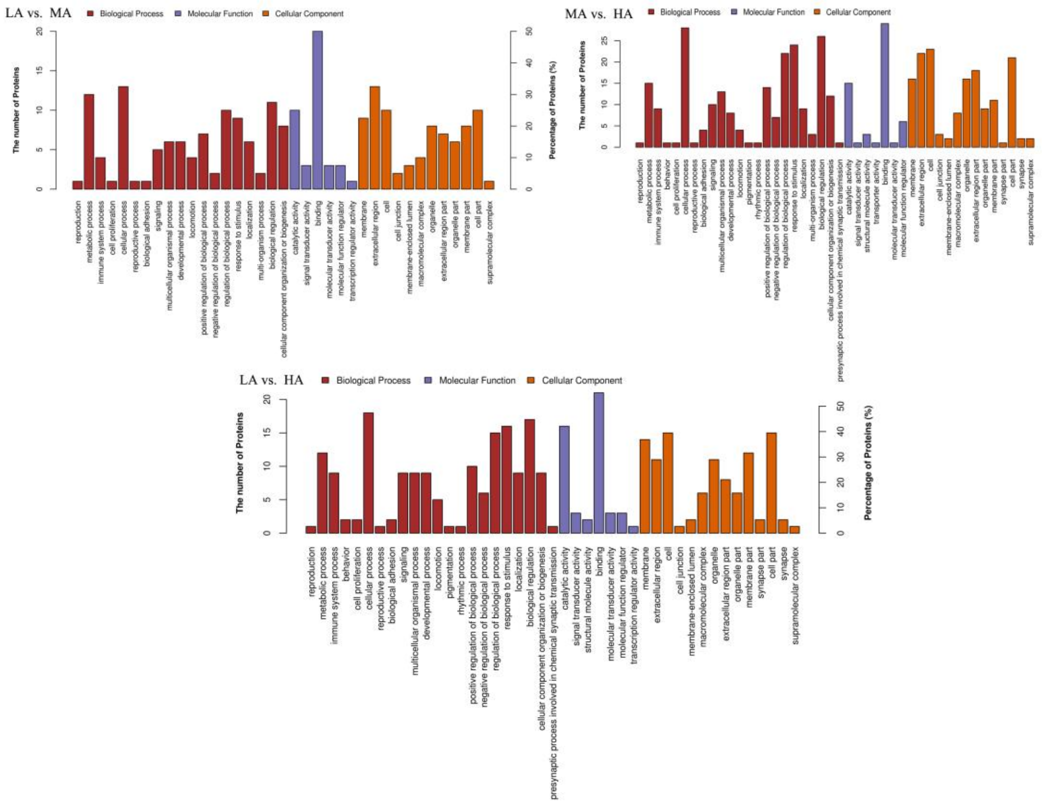

Figure. S3 Pairwise comparisons of the gene ontologies (GO) of the serum proteins between the three farms. LA, buffalo at low altitude; MA, buffalo at medium altitude; HA, buffalo at high altitude.
