# Supplementary material for: Physiological and Proteomic Responses of Dairy Buffalo to Heat Stress Induced by Different Altitudes
Source: Metabolites. 2022 Sep 27;12(10):909. doi: 10.3390/metabo12100909 (PMC9609643; doi:10.3390/metabo12100909)
Supplement: Supplementary file 1 [file metabolites-12-00909-s001.zip › Table S1 S2.pdf]

Table S1 Nutrient content of feed samples (%).

	Whole-plant corn silage	Concentrate feeding	Corn protein powder
Moisture content	73.64	8.65	8.30
EE	1.48	4.14	1.17
ASH	5.71	21.31	3.66
CP	7.56	40.18	67.22
Ca	0.30	3.29	0.18
P	0.20	1.08	0.54
ADF	35.25	15.48	8.97
ADICP	0.35	4.06	11.56
NDF	55.26	20.63	20.74
NDICP	0.24	6.18	5.98
ADL	4.95	4.34	3.30
NFC	30.24	19.92	13.19
tdNFC	29.63	19.52	12.93
tdNDF	30.97	3.15	4.84
tdCP	7.15	35.59	54.69
tdFA	0.48	3.14	0.17
DE_{1x}(Mcal/kg DM)	2.69	2.94	3.52
DE_{3x}(Mcal/kg DM)	2.62	2.92	3.36
ME_{3x}(Mcal/kg DM)	2.19	2.55	2.94
NE_{LP}(Mcal/kg DM)	1.35	1.60	1.88

CP: Crude protein; EE: Ether extract; Ca: Calcium; P: Phosphorus; ADF: Acid detergent fiber; NDF: Neutral detergent fiber; ADICP: Acid detergent insoluble crude protein; NDICP: Neutral detergent insoluble crude protein; ADL: Acid detergent lignin; NFC: Non-fiber carbohydrate; tdNDF: Truly digestible neutral detergent fiber; tdNFC: Truly digestible non-fiber carbohydrate; tdCP: Truly digestible crude protein; tdFA: Truly digestible fatty acids; DE1x: Digestible energy at one times maintenance level; DE3x: Digestible energy at three times maintenance level; ME3x: Metabolizable energy at three times maintenance level; NELP: Net energy for lactation at three times maintenance level; DM: Dry matter

Table S2 The same amount of proteins (μg) with different volumes (μl) for further analysis.

Serum samples	Concentration, μg/μl	20 μg	200 μg
LA1	10.1	2.0	19.8
LA2	12.1	1.7	16.5
LA3	10.6	1.9	18.9
LA4	9.7	2.1	20.7
LA5	9.9	2.0	20.1
LA6	11.3	1.8	17.7
LA7	8.2	2.4	24.3
LA8	9.1	2.2	21.9
LA9	12.2	1.6	16.5
MA1	11.5	1.7	17.4
MA2	9.7	2.1	20.7
MA3	10.7	1.9	18.7
MA4	8.9	2.2	22.4
MA5	11.1	1.8	18.0
MA6	9.8	2.0	20.5
MA7	12.1	1.6	16.5
MA8	11.4	1.8	17.5
MA9	10.4	1.9	19.3
MA10	9.7	2.1	20.7
MA11	10.7	1.9	18.7
MA12	9.3	2.2	21.6
HA1	12.4	1.6	16.2
HA2	12.0	1.7	16.7
HA3	12.1	1.7	16.6
HA4	10.4	1.9	19.2
HA5	11.2	1.8	17.9
HA6	11.5	1.7	17.4
HA7	12.9	1.6	15.5
HA8	10.5	1.9	19.1
HA9	12.8	1.6	15.7
HA10	13.3	1.5	15.1
HA11	11.6	1.7	17.2
HA12	11.1	1.8	18.1

The original samples were diluted by 3 times
